# Predictive factors for peripheral blood stem cell mobilization in multiple myeloma in the era of novel therapies: A single‐center experience

**DOI:** 10.1002/cam4.7356

**Published:** 2024-06-08

**Authors:** Xiao He, Duanfeng Jiang, Liang Zhao, Shuping Chen, Yan Zhu, Qun He, Yanjuan He

**Affiliations:** ^1^ Department of Hematology, Xiangya Hospital Central South University Changsha China; ^2^ National Clinical Research Center for Geriatric Disorders, Xiangya Hospital Central South University Changsha China; ^3^ Department of Hematology The Second Affiliated Hospital of Hainan Medical University Haikou China

**Keywords:** autologous transplantation, multiple myeloma, peripheral blood stem cells, poor mobilization, risk factors

## Abstract

**Objective:**

Multiple myeloma (MM) is the leading indication of autologous hematopoietic stem cell transplantation. The aim of this study was to determine the incidence of mobilization failure and characterize the risk factors associated with poor mobilization (PM) of MM patients in novel therapies era.

**Methods:**

We conducted a retrospective study of 211 MM patients who received their first peripheral blood stem cells (PBSC) mobilization at our single center. The following data were collected: age, gender, clinical stage, disease status, complete blood cell count, induction regimen, CD34^+^ cell count in peripheral blood (PB), and PBSC collections.

**Results:**

In addition to conventional drugs, 22 (10.4%) patients received daratumumab containing induction, and 33 (15.6%) patients used plerixafor for poor mobilization (pre‐apheresis PB CD34^+^ cells <20/μL). Failure of collection occurred in 24 (11.4%) patients and was correlated with low white blood cell (WBC), ≥3 cycles of lenalidomide treatment before mobilization, steady‐state mobilization and nouse of plerixafor are associated with mobilization failure. Daratumumab‐based induction treatment ≥2 courses, albumin >41 g/L before mobilization, and steady‐state mobilization were risk factors for PM in subgroups of patients treated with lenalidomide for <3 courses. In addition, Hepatitis B virus infection at baseline, thalassemia and measurable residual disease positivity were recognized as predictive factors for PM in subset of chemo‐mobilization patients.

**Conclusion:**

In addition to some well‐recognized risk factors, baseline WBC count and daratumumab exposure ≥2 courses before mobilization were revealed as the predictive factors of mobilization failure, providing consultation for preemptive use of plerixafor.

## 1 INTRODUCTION

Multiple myeloma (MM) is a malignant plasmacyte disease that tends to occur in the elderly.^1^ During the past few years, novel agents such as daratumumab, pomalidomide, and carfilzomib have increasingly been incorporated in first‐line induction therapies for MM.^2^, ^3^ Autologous hematopoietic stem cell transplantation (auto‐HCT) is also part of standard frontline therapy for most MM patients.^4^, ^5^ Nowadays, autologous peripheral blood stem cells (PBSC) have largely replaced bone marrow as the predominant source of stem cells in auto‐HCT in MM patients.^5^ Therefore, successful mobilization and stem cell collection is one of the prerequisites for auto‐HCT.^6^ Although conventional methods with the incorporation of novel agents such as plerixafor have been more successful in PBSC mobilization in MM, mobilization failure remains a worrying issue.^7^ Moreover, a minimum number of 2 × 10^6^ CD34^+^ cells/kg body weight and an optimal number of ≥5 × 10^6^ CD34^+^ cells/kg are required to ensure successful sustained hemopoietic recovery.^5^, ^8^, ^9^ However, the collection of sufficient autologous PBSC is based on various factors,^6^ and approximately 5% to 25% of patients are unable to collect enough cells. Although several risk factors have been identified,^5^, ^8^, ^10^ it is still challenging to predict mobilization failure in MM patients in the era of novel therapies.^5^, ^10^

Daratumumab‐based triplets or quadruplets are rapidly becoming the standard therapy in induction for MM.^2^, ^11^ However, concerns have arisen that daratumumab may be disadvantageous for PBSC mobilization. The impact of daratumumab‐based induction on PBSC mobilization has been evaluated in several clinical trials.^11^ In the CASSEIOPIA and GRIFFIN studies, patients treated with daratumumab‐based regimens were observed to be more dependent on plerixafor and had a higher apheresis days to achieve the target.^12^, ^13^ However, the specific mechanism of the effect of daratumumab on mobilization is still unclear. The novel agent plerixafor is a reversible CXCR4 inhibitor that improves PBSC mobilization.^14^ Plerixafor‐containing mobilization strategies have been previously shown to overcome the adverse effects of lenalidomide exposure and potentially lead to successful collection.^7^, ^15^, ^16^ Predicting the parameters for mobilization failure would allow for preemptive and more cost‐effective use of plerixafor during the first mobilization attempt; however, there are still a lot of unidentified risk factors for poor mobilization (PM).

In this study, we retrospectively collected clinical data on 211 patients with MM in our center from July 2019 to January 2023. The information collected included gender, age, bad habits (smoking or drinking alcohol), stage of the disease, baseline clinical indicators, comorbidities, induction treatment regimen, time from diagnosis to mobilization, disease status at the time of mobilization, and mobilization protocol. The intention of this study was to estimate the incidence of mobilization failure in MM patients in the era of novel therapies, as well as the predictive factors for PM in a single center.

## 2 MATERIALS AND METHODS

### 2.1 Study design and data collection

We retrospectively analyzed medical records of 211 MM patients, who underwent their first mobilization of PBSC at the Xiangya Hospital Bone Marrow Transplantation Center of Central South University between July 2019 and January 2023. Relevant information was obtained from electronic medical records. The following data elements were collected: age, gender, diagnosis, bad habits (smoking or drinking alcohol), time from diagnosis to mobilization, stage of the disease, baseline clinical indicators at diagnosis, comorbidities, treatment history, clinical indicators at mobilization, disease status at mobilization, and measurable residual disease (MRD) which was detected using multiparametric flow cytometry with a sensitivity of 10^−5^, mobilization protocol and collection results. In this study, drug exposure was defined as receiving for at least 1 cycle. For patients with multiple mobilization attempts, only the data from their first mobilization was included. This study was approved by the Medical Ethics Committee of Xiangya Hospital, Central South University, and was conducted in accordance with the principles of the Declaration of Helsinki.

### 2.2 Patients and induction treatment

The 211 patients included in this study were treated with VRD (bortezomib, lenalidomide, and dexamethasone), BCD (bortezomib, cyclophosphamide, and dexamethasone), PAD (bortezomib, doxorubicin liposomes, dexamethasone), DVRD (daratumumab, bortezomib, lenalidomide, and dexamethasone) and KCD (carfilzomib, cyclophosphamide, and dexamethasone) as the main induction therapy, and generally underwent mobilization after 3 to 4 cycles of induction therapy. A few patients postponed their mobilization plans due to infection, personal financial problems, and a long waiting time for hospitalization during the COVID‐19 pandemic. None of the patients had been infected with COVID‐19. All patients included in this study did not receive local radiotherapy before.

### 2.3 Stem cell mobilization and collection target

The mobilization regimens were divided into two modes: chemo‐mobilization and steady‐state mobilization. In both modes, salvage treatment with plerixafor (0.24 mg/kg) was given if the peripheral blood (PB) CD34^+^ cell count <20/μL. Stem cells were continuously collected for 1–3 days according to blood routine results and CD34^+^ cells obtained. The collection was considered successful when the number of CD34^+^ cells obtained was ≥2 × 10^6^/kg and failed when <2 × 10^6^/kg.

### 2.4 Chemo‐mobilization

Cyclophosphamide (CTX) was infused on 2 consecutive days (total dose, 3 g/m^2^). After CTX administration, patients can use 6 mg of long‐acting granulocyte‐colony stimulating factor (G‐CSF) after 48 h or short‐acting G‐CSF (10 μg/kg/day) from day 6. The monitoring of PB CD34^+^cell count began when PB white blood cell (WBC) count reached the lowest and then rise to 2 × 10^9^/L‐4 × 10^9^/L, or when the proportion of monocytes reached 20%–40%.

### 2.5 Steady‐state mobilization

Subcutaneous injection of G‐CSF (10 μg/kg/day) started from day 1, and the monitoring of PB CD34^+^cell count began on day 5.

### 2.6 Statistical analysis

Statistical analyses in this study were performed using SPSS version 24.0. Descriptive statistics was performed separately for the overall group and two groups of patients divided based on mobilization results. Univariate analysis was made using unpaired *t* test, Mann–Whitney U test or binomial logistic regression for continuous variables, the chi‐square test, Fisher exact test, or binomial logistic regression for categorical variables. Multivariate analysis was made using binomial logistic regression analysis and multiple linear regression to identify factors that influence mobilization results. The predictive capacity of the model was validated using the receiver operating characteristic curve (ROC) and the area under the curve (AUC). For all tests, values of *p* < 0.05 were considered statistically significant.

## 3 RESULTS

### 3.1 Patients and disease characteristics

A total of 211 patients were included in this study, and patient characteristics are listed in Table 1. Median age, gender, MM subtypes, stage at diagnosis, comorbidities, chemotherapy cycles received before mobilization, induction therapeutic agent, laboratory data at diagnosis and before mobilization, disease status at apheresis, MRD status, and mobilization protocols were compared for patients who had successful PBSC mobilization and those with PBSC mobilization failure. The median age of all patients at diagnosis was 56 years (range, 31–72). Among them, there were 125 males (59.2%) and 86 females (40.8%). At the time of diagnosis, 29 (13.7%), 143 (67.8%), and 39 (18.5%) patients were in stage I, II, and III of the revised International Staging System (R‐ISS), respectively. The median time from diagnosis to collection was 135 days (range, 70–1370). There were 194 (91.9%), 81 (38.4%), 28 (13.3%), 10 (4.7%), 22 (10.4%), and 10 (4.7%) patients exposed to bortezomib, lenalidomide, thalidomide, pomalidomide, daratumumab, and carfilzomib before mobilization, respectively. Steady‐state mobilization and chemo‐mobilization were used in 52 (24.6%) and 159 (75.4%) patients, respectively. Plerixafor was used in 33 patients (15.6%).

**TABLE 1:** Baseline patient characteristics and univariate analysis for CD34^+^ cell collection success and failure.

Characteristics /Predictive variables	Total (*n* = 211)	Success^a^ (*n* = 187)	Failure^b^ (*n* = 24)	OR (95% CI)	*p* Value
Male sex, *n* (%)	125 (59.2)	107 (57.2)	18 (75.0)	0.45 (0.17–1.17)	0.102
Age, year, median (IQR)	56 (50.61)	56 (50, 61)	57 (52, 65)	1.60 (0.68–3.75)	0.282
Smoking, *n* (%)	41 (19.4)	33 (17.6)	8 (33.3)	0.43 (0.17–1.08)	0.074
Alcohol use, *n* (%)	18 (8.5)	15 (8.0)	3 (12.5)	0.61 (0.16–2.29)	0.464
Time from diagnosis to collection, days (median, IQR)	135 (115, 150)	135 (115, 150)	139 (116.75, 160)	0.89 (0.38–2.08)	0.786
Subtype, *n* (%)					
IgG	102 (48.4)	91 (48.7)	11 (45.8)	1	
IgA	50 (23.7)	43 (23.0)	7 (29.2)	1.35 (0.49–3.72)	0.565
IgD	14 (6.6)	13 (7.0)	1 (4.2)	0.64 (0.08–5.34)	0.677
Light chain only	43 (20.4)	39 (20.9)	4 (16.7)	0.85 (0.25–2.83)	0.789
Non‐secretory	2 (0.9)	1 (0.5)	1 (4.2)	8.27 (0.48–141.81)	0.145
Light chain, *n* (%)					
κ	102 (48.3)	89 (47.6)	13 (54.2)	0.77 (0.33–1.80)	0.545
λ	107 (50.7)	97 (51.9)	10 (41.7)	1.51 (0.64–3.57)	0.349
ISS stage, *n* (%)					
I	39 (18.5)	34 (18.2)	5 (20.8)	1	
II	74 (35.1)	66 (35.3)	8 (33.3)	0.82 (0.25–2.71)	0.751
III	98 (46.4)	87 (46.5)	11 (45.8)	0.86 (0.28–2.66)	0.793
R‐ISS stage, *n* (%)					
I	29 (13.7)	25 (13.4)	4 (16.7)	1	
II	143 (67.8)	127 (67.9)	16 (66.7)	0.79 (0.24–2.55)	0.691
III	39 (18.5)	35 (18.7)	4 (16.7)	0.71 (0.16–3.13)	0.655
Comorbidities, *n* (%)					
Diabetes	17 (8.1)	16 (8.6)	1 (4.2)	2.15 (0.27–17.0)	0.467
Hypertension	41 (19.4)	37 (19.8)	4 (16.7)	1.23 (0.40–3.83)	0.717
Hepatitis B	13 (6.2)	9 (4.8)	4 (16.7)	0.25 (0.07–0.90)	0.033
Thalassemia	5 (2.4)	3 (1.6)	2 (8.3)	0.18 (0.03–1.13)	0.068
Other tumors	6 (2.9)	4 (2.1)	2 (8.3)	0.24 (0.04–1.39)	0.111
Baseline^c^					
WBC, median (IQR)	5.7 (4.2, 6.7)	5.8 (4.5, 6.9)	4.0 (3.4, 5.4)	0.16 (0.04–0.56)	<0.001
Hb, median (IQR)	97 (78, 112)	97 (79, 114)	87.5 (68, 104)	0.43 (0.17–1.07)	0.070
PLT, median (IQR)	192 (132, 231)	194 (141, 235)	155 (109, 212)	0.46 (0.19–1.14)	0.046
SCr, median (IQR)	87.5 (71, 124)	87.5 (70, 133)	86.0 (75, 100)	0.69 (0.29–1.62)	0.394
Alb, median (SD)	35.63 (6.38)	35.57 (6.27)	36.06 (7.29)	0.41 (0.96–2.27)	0.317
Ca^2+^, median (SD)	2.42 (0.38)	2.43 (0.38)	2.33 (0.34)	0.66 (0.25–1.74)	0.396
B2M, median (IQR)	4.7 (3.3, 7.6)	4.7 (3.3, 7.6)	4.7 (2.8, 9.9)	1.55 (0.66–3.63)	0.317
LDH (median, IQR)	178.3 (150, 275)	178.3 (149, 214)	178.4 (155, 224)	1.33 (0.56–3.14)	0.519
Before mobilization					
Alb, median (SD)	41.49 (3.40)	41.37 (3.28)	42.47 (4.20)	0.86 (0.77–4.45)	0.166
Ca^2+^, median (SD)	2.26 (0.11)	2.26 (0.11)	2.27 (0.11)	1.00 (0.42–2.33)	0.990
B2M, median (IQR)	2.9 (2.1, 3.9)	3.0 (2.1, 3.9)	2.7 (2.1, 3.3)	0.60 (0.25–1.42)	0.247
LDH, median (IQR)	200 (173, 233)	200 (173, 236)	197 (166, 223)	0.80 (034–1.88)	0.602
D‐dimer, median (IQR)	0.17 (0.09, 0.31)	0.17 (0.09, 0.31)	0.15 (0.08, 0.27)	0.79 (0.34–1.86)	0.595
Prior chemotherapy cycles, median (IQR)	4 (3,4)	3 (3,4)	4 (3,4)	0.56 (0.23–1.33)	0.189
Cycles of lenalidomide, *n* (%)					
<3	167 (79.1)	153 (81.8)	14 (58.3)	1	
≥3	44 (20.9)	34 (18.2)	10 (41.7)	3.21 (1.32–7.85)	0.010
Cycles of daratumumab, *n* (%)					
<2	195 (92.4)	176 (94.1)	19 (79.2)	1	
≥2	16 (7.6)	11 (5.9)	5 (20.8)	4.21 (1.32–13.41)	0.015
Use of thalidomide, *n* (%)	28 (13.3)	27 (14.4)	1 (4.2)	3.88 (0.50–29.95)	0.193
Use of pomalidomide, *n* (%)	10 (4.7)	10 (5.3)	0 (0.0)	‐	
Use of carfilzomib, *n* (%)	10 (4.7)	9 (4.8)	1 (4.2)	1.16 (0.14–9.60)	0.889
Disease status at apheresis, *n* (%)					
≥ VGPR	163 (77.3)	148 (79.1)	15 (62.5)	1	
<VGPR	48 (22.7)	39 (20.9)	9 (37.5)	2.28 (0.93–5.59)	0.073
MRD positivity, *n* (%)	83 (39.3)	71 (38.0)	12 (50.0)	0.61 (0.26–1.44)	0.259
Mobilization regimen, *n* (%)					
Chemo‐mobilization	159 (75.4)	148 (79.1)	11 (45.8)	1	
Steady‐state	52 (24.6)	39 (20.9)	13 (54.2)	4.49 (1.87–10.78)	<0.001
Use of plerixafor, *n* (%)	33 (15.6)	30 (16.0)	3 (12.5)	1.34 (0.38–4.77)	0.654

^a^ Successful mobilization, defined as a CD34^+^ cell count ≥2 × 10^6^ /kg of recipient weight per intended transplant.

^b^ Mobilization failure, defined as a CD34^+^ cell count <2 × 10^6^ /kg of recipient weight per intended.

^c^ Baseline means at time of diagnosis, before induction therapy.

Abbreviation: Alb, albumin; B2M, β_2_‐microglobulin; CI, confidence interval; Hb, hemoglobin; ISS, International Staging System; IQR, interquartile range; LDH, lactic dehydrogenase; MRD, minimal residual disease; OR, odds ratio; PLT, platelet; RISS, Revised International Staging System; SCr, Serum creatinine; SD, standard deviation; VGPR, very good partial remission; WBC, white blood cell.

### 3.2 Predictors of stem cell mobilization failure by univariate and multivariate analysis

**FIGURE 1:**
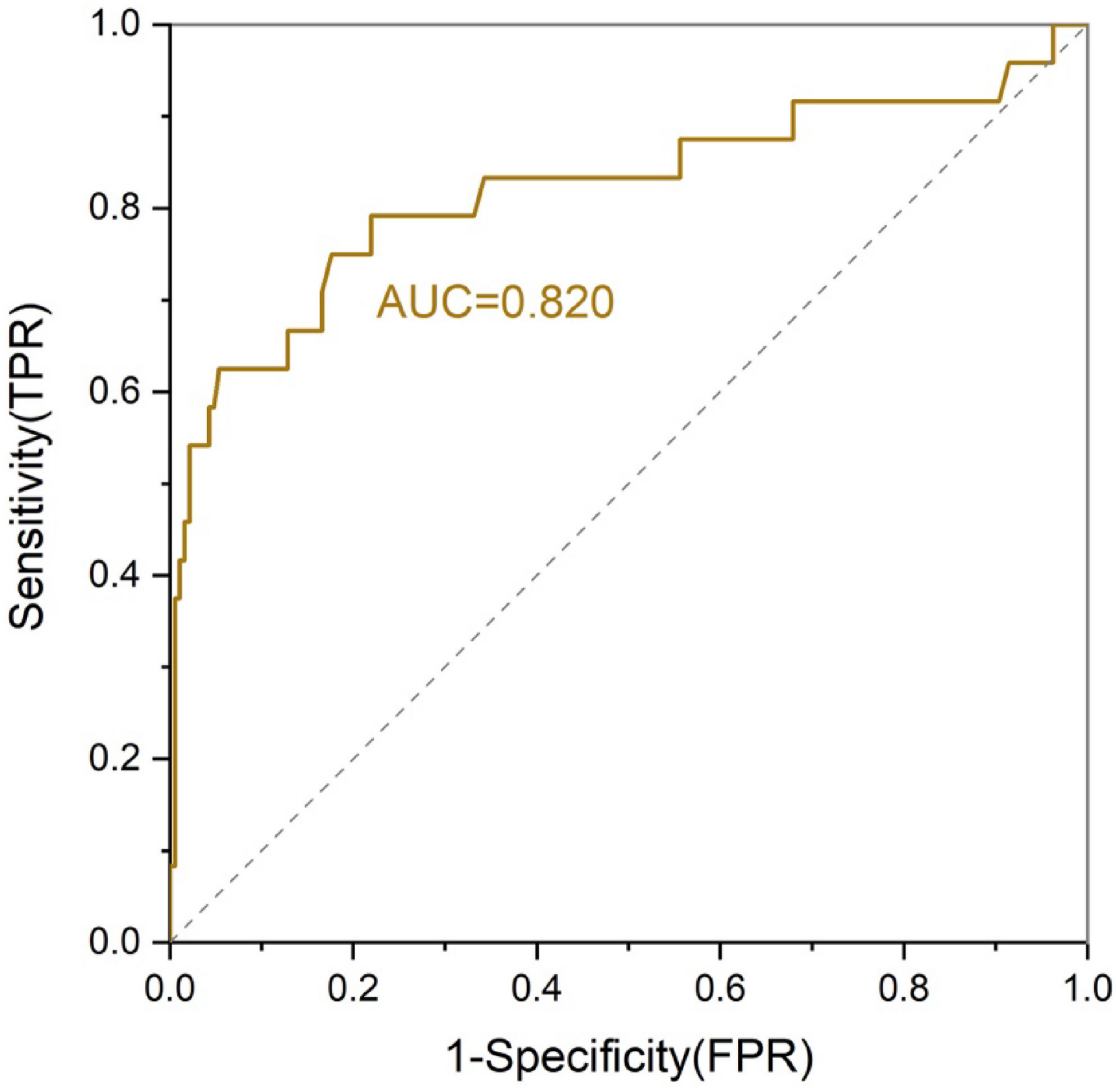
Receiver operating characteristic (ROC) curve of predictive model for failure of mobilization. ROC curve of the prediction model based on the four independent risk factors (baseline WBC count <5.7 × 10^9^/L, lenalidomide treatment ≥3 cycles, steady‐state mobilization and nonuse of plerixafor). The ROC area under the curve (AUC) to the outcome of PM was 0.82 (95% CI: 0.73–0.94, *p* < 0.001).

We used a logistic regression to identify potential risk factors for mobilization failure. Overall, The median number of collected CD34^+^ cells was 5.53 (interquartile range 0.14–57.4) × 10^6^ cells/kg, and 24 patients (11.4%) failed to successfully mobilize a sufficient number of stem cells (2 × 10^6^ CD34^+^ cells/kg) with the first collection attempt. In the univariate analysis, WBC count <5.7 × 10^9^/L (failure rate: 16.9% vs. 4.3%; OR 0.25, *p* < 0.001), lower platelet count <192 × 10^9^/L (failure rate: 17.0% vs. 5.7%; OR 0.46, *p* = 0.046), and Hepatitis B virus (HBV) infection (failure rate: 30.8% vs. 11.2%; OR 0.16, *p* = 0.033) at baseline, ≥3 cycles of lenalidomide exposure (failure rate: 22.7% vs. 8.4%; OR 3.21, *p* = 0.010) before mobilization and steady‐state mobilization (failure rate: 25.0% vs. 6.9%; OR 4.49, *p* < 0.001) were associated with failure to collect sufficient PBSC (Table 1). It is worth noting that patients who received ≥2 cycles of dalatuzumab containing induction therapy had a higher proportion of mobilization failure (31.3% vs. 9.7%; OR 4.49, *p* = 0.015). By multivariate analysis using the six parameters identified by univariate analysis, baseline WBC count <5.7 × 10^9^/L (adjusted OR 0.57, 95% CI: 0.40–0.81, *p* = 0.002) was identified as a risk factor for PM. Consistent with previous studies, ≥3 cycles of lenalidomide treatment (adjusted OR 3.45, 95% CI: 1.25–9.56, *p* = 0.017), steady‐state mobilization (adjusted OR 9.45, 95% CI: 3.14–28.44, *p* < 0.001), and nonuse of plerixafor (adjusted OR 0.15, 95% CI: 0.03–0.72, *p* = 0.018) were the significant predictors of failure to collect PBSC after mobilization (Table 2). Receiver operating characteristic (ROC) curves were generated to determine the predictive value of risk factors. We developed a new ROC curve of the prediction model by assigning one point for each of the four independent predictors (baseline WBC count <5.7 × 10^9^/L, lenalidomide treatment ≥3 cycles, steady‐state mobilization and nonuse of plerixafor). As shown in Figure 1, the ROC area under the curve (AUC) to the outcome of PM was 0.82 (95% CI: 0.73–0.94, *p* < 0.001).

**TABLE 2:** Multivariate analysis of risk factors associated with mobilization failure.

Variables	OR	95%CI	*p* Value
Baseline WBC <5.7 × 10^9^/L	0.57	0.40–0.81	0.002
Lenalidomide exposure ≥3 cycles	3.45	1.25–9.56	0.017
Steady‐state mobilization	9.45	3.14–28.44	<0.001
No‐use of plerixafor	0.15	0.03–0.72	0.018

Abbreviations: CI, confidence interval; OR, odds ratio.

### 3.3 Subgroup analysis of patients mobilized by cyclophosphamide

Due to the significant effect of steady‐state mobilization on statistical result, a subgroup analysis of the patients underwent chemo‐mobilization was performed to explore risk factors for PM. Eleven patients (11/159, 6.9%) failed to provide sufficient CD34^+^ cells (<2 × 10^6^ cells/kg). In the univariate analysis, three variables were identified as risk factor for PM, including HBV infection (adjusted OR 11.12, 95% CI: 2.09–59.25, *p* = 0.005), thalassemia (adjusted OR 16.67, 95% CI: 1.82–152.97, *p* = 0.013), and MRD positivity (adjusted OR 4.41, 95% CI: 1.02–19.11, *p* = 0.047) (Table 3). Furthermore, exposure to lenalidomide was no longer a risk factor for mobilization failure in the chemo‐mobilization subgroup, which is consistent with previous reports.^17^, ^18^

**TABLE 3:** Subset analysis of chemo‐mobilization patients (*N* = 159).

Variables	Univariate analysis	Multivariate analysis		
	*p* Value	OR	95% CI	*p* Value
Male sex	0.252			
Age >56	0.976			
Hepatitis B	0.020	11.12	2.09–59.25	0.005
Thalassemia	0.024	16.67	1.82–152.97	0.013
Baseline WBC <5.4 × 10^9^/L	0.044			
Prior chemotherapy cycles >4	0.860			
≥3 lenalidomide	0.316			
≥2 daratumumab	0.610			
Use of plerixafor	0.614			
MRD positivity	0.040	4.41	1.02–19.11	0.047
<VGPR	0.382			

Abbreviations: CI, confidence interval; MRD, minimal residual disease; OR, odds ratio; VGPR, very good partial remission; WBC, white blood cell.

### 3.4 Subgroup analysis of patients exposed to lenalidomide less than 3 cycles

Longer exposure to lenalidomide has a negative impact on stem cell yield.^19^ Due to the significant effect of ≥3 cycles of lenalidomide on outcome of mobilization showing as above data, we screened patients with <3 cycles of lenalidomide for subgroup analysis to further explore other potential risk factors. Of the 167 patients screened, 14 patients (8.4%) failed mobilization. The results showed that Baseline WBC count <5.4 × 10^9^/L (*p* = 0.015), albumin >41 g/L before mobilization (*p* = 0.017), ≥ 2 cycles of daratumumab exposure in prior induction (*p* = 0.007), and steady‐state mobilization (*p* = 0.040) were related with collection failure in univariate analysis. However, only ≥2 cycles of daratumumab exposure in prior induction (adjusted OR 6.58, 95% CI: 1.49–29.13, *p* = 0.013), albumin >41 g/L before mobilization (adjusted OR 1.21, 95% CI: 1.03–1.44, *p* = 0.025), and steady‐state mobilization (adjusted OR 3.37, 95% CI: 1.02–11.09, *p* = 0.046) were statistically significant in multivariate analysis (Table 4). It is worth noting that most patients (167/211, 79.1%) in our center were not exposed to lenalidomide for more than 2 cycles, and daratumumab was identified as a risk factor for mobilization failure in this group of patients.

**TABLE 4:** Subset analysis of patients treated with lenalidomide for less than 3 cycles (*N* = 167).

Variables	Univariate analysis	Multivariate analysis		
	*p* Value	OR	95%CI	*p* Value
Male sex	0.071			
Age >56	0.679			
Prior chemotherapy cycles >4	0.583			
Baseline WBC <5.4 × 10^9^/L	0.015			
Alb before mobilization >41 g/L	0.017	1.21	1.03–1.44	0.025
≥2 daratumumab	0.007	6.58	1.49–29.13	0.013
<VGPR	0.652			
Use of plerixafor	0.684			
Steady‐state	0.040	3.37	1.02–11.09	0.046

Abbreviation: Alb, albumin; CI, confidence interval; OR, odds ratio; VGPR, very good partial remission; WBC, white blood cell.

### 3.5 Impact of daratumumab‐based induction on stem cell collection

**FIGURE 2:**
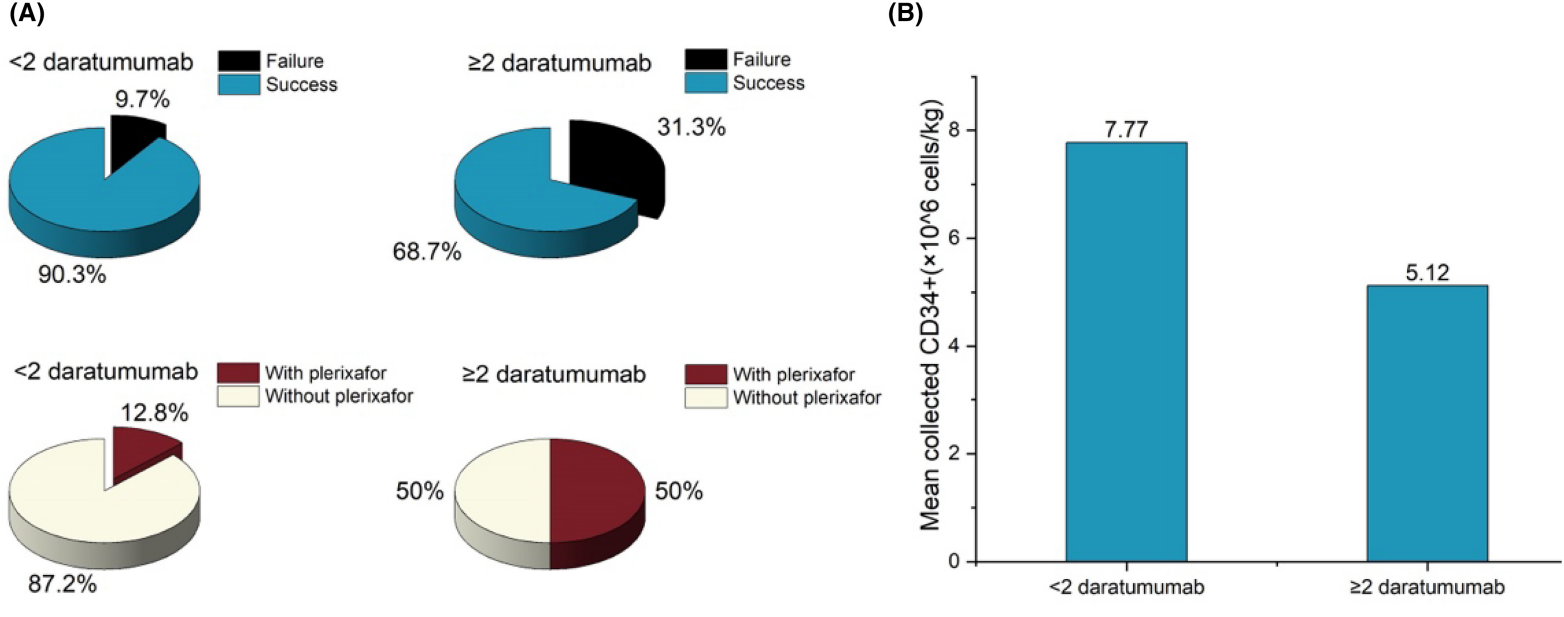
< 2 cycles of daratumumab versus ≥2 cycles of daratumumab. (A) The pie graphs in the first row show that the rate of collection failure in MM patients with ≥2 cycles of daratumumab treatment before mobilization was higher than that <2 cycles of daratumumab (31.3% vs. 9.7%, *p* = 0.015). The pie graphs in the second row show that MM patients with ≥2 cycles of daratumumab treatment before mobilization required more frequent use of plerixafor than that <2 cycles of daratumumab (50.0% vs. 12.8%, *p* < 0.001). (B) The bar chart shows MM patients with ≥2 cycles of daratumumab treatment before mobilization had lower peripheral blood CD34^+^ cells collection than that <2 cycles of daratumumab (mean 5.12 × 10^6^ vs. 7.77 × 10^6^ cells/kg, *p* = 0.049).

Exposure to daratumumab before collection was not found to impact the rate of mobilization failure.^6^ However, recent studies have shown that the exposure of daratumumab during induction leads to lower stem cell yield and may have a negative effect on stem cell mobilization and collection.^11^, ^20^ In our preliminary study, we observed that patients who use daratumumab are more dependent on plerixafor. To explore the correlation, we grouped patients by whether they had used ≥2 cycles of daratumumab or not before mobilization. Among the whole‐cohort patients, the patients in the ≥2 cycles of daratumumab group were more dependent on plerixafor than the control group (8/16, 50.0% vs. 25/195, 12.8%; *p* < 0.001), and had a higher mobilization failure rate than the control group (5/16, 31.3% vs. 19/195, 9.7%; *p* = 0.015) (Figure 1). Moreover, exposure of daratumumab ≥2 cycles during induction leads to statistically significant lower stem cell yield (mean 5.12 × 10^6^ vs. 7.77 × 10^6^ cells/kg, *p* = 0.049) (Figure 2).

### 3.6 Predictors of stem cell collection efficiency by univariate and multivariate analysis

Due to the potential impact of different physician practices and up‐front stem cell collection targets, we investigated the effects of the various baseline and treatment factors on collection efficiency. Univariate analysis showed that Baseline WBC count <5.4 × 109/L (*p* = 0.044), lenalidomide exposure (*p* = 0.033), Steady‐state mobilization (*p* < 0.001), and mobilization with no plerixafor (*p* = 0.021) were associated with lower stem cell collection efficiency. By multivariate analysis, only steady‐state mobilization was independently associated with poor efficiency (adjusted *p* < 0.001; Table 5).

**TABLE 5:** Uni‐ and multi‐variate analysis of factors affecting stem cell collection efficiency.

Variables	Efficiency median (IQR)	Coefficient estimate	*p* Value	Adjusted coefficient estimate	Adjusted *p* Value
Hepatitis B					
No	6.0 (3.4, 9.9)	0	0.119		
Yes	4.7 (1.0, 8.4)	−1.974			
Baseline WBC					
≥5.4 × 10^9^/L	5.1 (3.0, 8.9)	0	0.044	0	0.219
<5.4 × 10^9^/L	6.7 (3.6, 10.9)	0.293		0.177	
Baseline PLT					
≥150 × 10^9^/L	6.2 (3.6, 10.5)	0	0.083		
<150 × 10^9^/L	4.9 (2.7, 8.5)	0.007			
Baseline Alb					
≥35 g/L	6.1 (3.6, 10.3)	0	0.065		
<35 g/L	5.5 (3.1, 9.1)	0.091			
Time from diagnosis to collection					
≤135 days	6.0 (3.5, 9.1)	0	0.114		
>135 days	5.3 (3.3, 10.3)	−0.005			
Prior chemotherapy cycles					
≤4	6.1 (3.6, 9.9)	0	0.25		
>4	3.5 (3.0, 6.2)	−0.171			
Use of lenalidomide					
No	7.1 (4.0, 11.6)	0	0.033	0	0.211
Yes	4.4 (2.7, 8.1)	−1.367		−0.795	
Use of daratumumab					
No	6.2 (3.6, 10.2)	0	0.069		
Yes	3.0 (2.0, 4.9)	−1.932			
Use of pomalidomide					
No	6.1 (3.5, 10.0)	0	0.155		
Yes	2.9 (2.5, 4.0)	−2.039			
Use of carfilzomib					
No	6.1 (3.6, 10.2)	0	0.097		
Yes	2.8 (2.1, 5.2)	−2.377			
<VGPR					
No	6.2 (3.6, 10.3)	0	0.261		
Yes	4.2 (2.8, 10.2)	−0.85			
MRD positivity					
No	6.3 (3.6, 10.7)	0	0.509		
Yes	5.6 (3.3, 10.9)	−0.426			
Steady‐state					
No	7.3 (4.4, 11.6)	0	<0.001	0	<0.001
Yes	3.3 (2.5, 4.8)	−3.273		−3.209	
Use of plerixafor					
No	6.7 (3.6, 10.9)	0	0.021	0	0.571
Yes	4.0 (2.8, 6.4)	−1.942		0.582	

Abbreviations: CI, confidence interval; IQR, interquartile range; MRD, minimal residual disease; OR, odds ratio; VGPR, very good partial remission; WBC, white blood cell.

## 4 DISCUSSION

In this study, a total of 211 adult patients with MM were retrospectively included for identifying the incidence of mobilization failure and the predictive factors for PM. Data from our center showed a rate of 11.4% patients failed to collect more than or equal to 2 × 10^6^ CD34^+^ cells/kg at their first stem cell collection attempt. We have identified that low WBC, ≥3 cycles of lenalidomide treatment before mobilization, steady‐state mobilization and nonuse of plerixafor are associated with mobilization failure. Using the four risk factors identified in this study, we created a risk model that may predict PM before stem cell mobilization.

Corso et al.^21^ and Pozotrigo et al.^22^ have reported that low WBC count, low platelet count, and steady‐state mobilization (use of single‐agent G‐CSF for mobilization) were associated with a lower number of CD34^+^ cells collected and PM, which is similar to our findings. Notably, the patients in these studies were treated mainly with chemotherapy or traditional targeted drugs. The patients in our study received both novel agent‐ and chemotherapy‐based regimens, suggesting that these predictive factors can be applied to novel agent‐based therapies. Previous studies have reported prior lenalidomide use was significantly associated with failed mobilization.^23^, ^24^, ^25^ Dosani et al.^26^ found exposure to lenalidomide did not impact PBSC collection outcome due to high plerixafor use (70%), indicating that the negative effect of lenalidomide exposure on PBSC collection can be attenuate by plerixafor‐based mobilization. Consistently, our data showed that ≥3 cycles of lenalidomide treatment before mobilization is correlated with poor mobilization in patients with no plerixafor‐based mobilization. Previous studies have reported that counting PB CD34^+^ cells can predict PM before collecting PBSC.^27^, ^28^ Olivieri et al.^10^ created a ROC curve by using premobilization low WBC count, low platelet count, use of G‐CSF alone (steady‐state mobilization), and not providing up‐front plerixafor, with the ROC curve (area under the curve 0.80, 95% CI: 0.76–0.84). In comparison, our models have a high sensitivity and specificity for predicting PM. Although some of the previously reported risk factors such as advanced age, smoking, and diabetes were not statistically significant in our study.

It is worth noting that, despite the above risk factors in whole‐cohort patients analysis are similar to previous studies,^5^, ^8^, ^9^, ^23^, ^29^, ^30^, ^31^ other predictive factors were revealed in this study. Exposure to daratumumab for ≥2 cycles prior mobilization and HBV infection at baseline were found to be associated with failure of stem cell collection in the univariate analysis of whole‐cohort patients. These two variables were validated as risk factors for PM in the multivariate analysis of subgroups of patients treated with lenalidomide for <3 cycles and subset of chemo‐mobilization patients, respectively. Moreover, high albumin level before mobilization was predictive factors for PM in subgroups of <3 cycles lenalidomide. Thalassemia and MRD positivity were identified as risk factors for PM in subset of chemo‐mobilization.

Ozkurt et, al.^32^ reported serum ferritin levels were negatively correlated with PB CD34^+^ cell count the first mobilization. Van Timothee et, al.^33^ found the iron overload was associated with poorer mobilization of PBSC in pediatric patients with thalassemia. Consistently, our study investigated that thalassemia was related to PM in patients of chemo‐mobilization, and thalassemia patients in this study had high levels of serum ferritin (data not shown). Previous studies have found that albumin played a negative role in the distribution of G‐CSF in bone marrow, providing a reason for poor mobilization efficiency.^34^ The prevalence of HBV infection in our study was 7.1%. Li et, al.^35^ reported the HBsAg^+^ status is an independent risk factor for patients with MM receiving auto‐HCT. However, this is the first study reveal that albumin >41 g/L before mobilization, HBV infection and MRD positivity maybe predictive factors for PM in patients with MM, and more studies are needed to clarify these findings in future.

As novel drugs, such as daratumumab, pomalidomide, and carfilzomib, are increasingly incorporated into the first‐line induction therapies for MM,^2^, ^3^ traditional risk factors for PM are different. CASSEIOPIA, GRIFFIN, and several other phase 3 studies have supported that the daratumumab‐based triplet or quadruplet regimen as the first‐line therapy in induction.^12^, ^13^, ^36^, ^37^ In this study, 22 (10.4%) of the patients received daratumumab containing induction. The patients who underwent daratumumab‐based induction treatment for ≥2 cycles had a higher proportion of collection failures than the control (31.3% vs. 9.7%). Moreover, the administration of ≥2 cycles of standard‐dose daratumumab was found to be an independent risk factor for mobilization failure in patients <3 cycles of lenalidomide exposure. To collect sufficient CD34^+^ cells, patients exposed ≥2 cycles of daratumumab treatment had a higher rate of up‐front use of plerixafor in our study. Recently, several studies have reported that daratumumab‐based induction regimens can lead to poor mobilization.^11^, ^21^ The randomized phase III CASSIOPEIA study,^12^ the GRIFFIN and MASTER trials^11^ as well as in small retrospective series^38^ showed that, daratumumab‐based induction group patients required more frequent use of plerixafor, larger collection volumes, and had lower stem cell yield. However, another study found that there was no difference in the total number of CD34^+^ cells collection between the DVRD and VRD groups if patients were preemptively treated with plerixafor.^11^, ^39^, ^40^ These results are consistent with our data, which suggested that daratumumab is associated with PM in the context of intervention without the use of plerixafor. However, we know little about the molecular mechanisms by which daratumumab affects mobilization. A report indicated that elevated serum levels of anti‐CD38 monoclonal antibody may affect mobilization by binding to low levels of CD38 antigen expression of CD34^+^ cells.^2^

Predicting mobilization failure before it starts may enable patients to develop tailored strategies, such as omitting CTX in induction, monitor closely and use early plerixafor in case of low PB CD34^+^ cells count. It has been suggested that the negative effect of lenalidomide on mobilization can be overcome by chemo‐mobilization.^17^, ^24^ In addition, our center favors the priority use of chemo‐mobilization for all patients since steady‐state mobilization was identified as independent risk factor. The preemptive use of plerixafor appeared to overcome the negative impact of daratumumab‐based induction on stem cell yield. However, the strategies of plerixafor using for stem cell mobilization was different during institutional practice, including up‐front (i.e., planned plerixafor use according to PM risk factors) or rescue (i.e., plerixafor use only after mobilization failure with G‐CSF alone). Up‐front plerixafor strategy may be considered, which was reported superior to rescue plerixafor.^11^, ^29^ The strategy of preemptive use of plerixafor along with G‐CSF has been adopted by our institution to allow for more efficient collection.

In this study, in addition to some well‐recognized risk factors, we have also revealed several little‐reported factors that we hope will draw more attention and help to obtain more effective ways to avoid mobilization failures in the future. Knowing more about the factors that predict mobilization failure would allow for a preemptive and more cost‐effective use of novel agents in the first mobilization attempt. We also presented the experience of plerixafor mobilization for the poor mobilizers related to daratumumab exposure.

## 5 CONCLUSIONS

In summary, mobilization failure occurred in 11.4% of patients overall. We suggest that baseline WBC count and daratumumab exposure ≥2 cycles before mobilization are the predictive factors of mobilization failures. Observation of baseline WBC count would be helpful to consider further mobilization approaches, such as chemo‐mobilization and up‐front use of plerixafor. In addition to adjusting mobilization strategies, the successful collection of sufficient number of CD34^+^ cells depends on many factors. Hence these mobilizing strategies should be individualized before initiating the treatment plan for an auto‐HCT.

## AUTHOR CONTRIBUTIONS

**Xiao He:** Data curation (lead); formal analysis (lead); methodology (lead); software (equal); writing – original draft (equal). **Duanfeng Jiang:** Formal analysis (equal); methodology (equal); software (equal); writing – original draft (equal). **Liang Zhao:** Data curation (supporting); formal analysis (supporting); methodology (supporting); software (equal); writing – original draft (supporting). **Shuping Chen:** Conceptualization (equal); project administration (equal); resources (lead); supervision (equal). **Yan Zhu:** Conceptualization (equal); investigation (equal); project administration (equal); resources (equal); visualization (equal). **Qun He:** Conceptualization (supporting); project administration (equal); resources (supporting); supervision (equal). **Yanjuan He:** Conceptualization (lead); investigation (lead); project administration (lead); resources (supporting); visualization (lead); writing – review and editing (lead).

## FUNDING INFORMATION

The authors declare that no funds, grants, or other support were received during the preparation of this manuscript.

## CONFLICT OF INTEREST STATEMENT

The authors have no relevant financial or nonfinancial interests to disclose.

## ETHICS STATEMENT

This study was approved by the Medical Ethics Committee of Xiangya Hospital, Central South University, and was conducted according to the principles of the Declaration of Helsinki.

## Data Availability

The data of this study are not publicly available because the information can compromise the privacy of research participants. The datasets generated and/or analyzed during the current study are available from the corresponding author on reasonable request.
